# Effect of the isotiazole adjuvants in combination with cisplatin in chemotherapy of neuroepithelial tumors: experimental results and modeling

**DOI:** 10.1038/s41598-023-40094-9

**Published:** 2023-08-21

**Authors:** Vladimir Potkin, Aliaxandr Pushkarchuk, Alexandra Zamaro, Hongwei Zhou, Sergey Kilin, Sergey Petkevich, Irina Kolesnik, Dominik L. Michels, Dmitry A. Lyakhov, Vladimir A. Kulchitsky

**Affiliations:** 1 Laboratory of the Chemistry of Heterocyclic Compounds, Institute of Physical Organic Chemistry, National Academy of Sciences of Belarus, 13 Surganov Str., 220072 Minsk, Belarus; 2 Brain Centre, Institute of Physiology, National Academy of Sciences of Belarus, 28 Akademicheskaya Str., 220072 Minsk, Belarus; 3 College of Biological, Chemical Sciences and Engineering, Jiaxing University, 1 Jiahang Road, Jiaxing, 314001 Zhejiang China; 4 Center “Quantum Optics and Quantum Informatics”, B.I. Stepanov Institute of Physics, National Academy of Sciences of Belarus, 68 Nezavisimosti Ave., 220072 Minsk, Belarus; 5 Computer, Electrical and Mathematical Science and Engineering Division, King Abdullah University of Science and Technology, Thuwal, 23955-6900 Kingdom of Saudi Arabia

**Keywords:** Chemotherapy, Cheminformatics

## Abstract

Chemotherapy is one of the main treatment options for cancer, but it is usually accompanied with negative side effects. The classical drugs combination with synergistic adjuvants can be the solution to this problem, allowing reducing therapeutic dose. Elucidating the mechanism of adjuvant action is of key importance for the selection of the optimal agent. Here we examine the system drug-adjuvant to explain the observed effect in practice. We used the first line drug cisplatin. Morpholinium and 4-methylpiperazinium 4,5-dichloro isothiazol-3-carboxylates were selected as adjuvants. The study of the cisplatin-adjuvant system was carried out by quantum chemical modeling using DFT. It turned out that adjuvants form conjugates with cisplatin that lead to the relocation of frontier molecular orbitals as well as increase of conjugate’s dipole moment. It resulted in change of the interaction character with DNA and increase of the bioactivity of the system. The data obtained are the basis for expanding the studies to include other drugs and adjuvants. Oncologists will have opportunity to use “classical” chemotherapy drugs in combination with synergists for those patients who have not been previously recommended to such a treatment because of pronounced toxic side effects.

## Introduction

The problem of radical treatment of malignant neoplasms has not yet been resolved^1^. Three main methods dominate in clinical oncology: surgical removal of tumor, radiation therapy and chemotherapy^2^. All these methods are often used in different combinations to improve treatment effectiveness. Our research focuses on one of these approaches—chemotherapy, which still remains the main method of cancer treatment. The choice of this particular section of clinical oncology is based on significant progress in creation of anticancer chemotherapy drugs, especially in those nosological forms of tumors when it is impossible to use surgical or radiation methods^3^. Successful use of chemotherapy is hindered by a number of factors; coping with them will increase effectiveness of anticancer therapy and reduce mortality in oncology. Nonselective toxicity of used substances causing fatal damage to both tumor and all other rapidly proliferating cells in patient's body is one of the main disadvantages of chemotherapy^4^. In this regard, improvement of tumor chemotherapy includes both the search for new effective antineoplastic targeted agents and development of ways to reduce side effects, primarily toxicity of anticancer therapy. International protocols recommend limiting single and total dose as well as the number of courses to weaken side effects of chemotherapy drugs, but compliance with these conditions is accompanied with natural weakening of anticarcinogenic effects and decrease in treatment effectiveness. The use of synergistic adjuvants in combined chemotherapy of tumors in order to enhance antitumor effects and reduce therapeutic dosages of official chemotherapy drugs is one of the solutions to this problem. Such tactics are aimed at keeping recognized chemotherapy drugs in the list as their development is extremely expensive, and also at protecting patient's organism weakened by tumor process from side effects of cytostatics.

The authoring team of the article has been conducting research on molecular design of structures, synthesis and biotesting of new synergistic adjuvants in compositions with first-line anticancer drugs for a number of years. As a result, it was found that isothiazole derivatives are promising candidates for synergist’s role. Isothiazole (1,2-thiazole) nucleus has high potential of biological activity and is the structural part of a wide range of synthetic bioactive substances^5^.

We found that many isothiazole derivatives have potentiating effect in binary mixtures with bioactive compounds, including antitumor agents^6–8^. These are represented by 4,5-dichloroisothiazole-3-carboxylic acid, its salts, amides, esters, carbamates, urea, metal complexes with 4,5-dichloroisothiazole moieties, etc. The observed potentiating effect is so pronounced in some cases that it makes possible to reduce the dose of chemotherapy drug by a decade in in vitro experiments on C6 glioma culture cells or on primary culture cells from patient neuroepithelial tumor. Thus, an experimental substantiation of methodology is being formed for creating a new generation of anticancer drugs based on combination of existing chemotherapy substances with adjuvants recommended in the process of studies^9^.

It is advisable to identify key factors that determine synergistic effect of an adjuvant in a binary mixture, i.e., the mechanism of its manifestation at the molecular level for the purposeful development of new synergists and their compositions with anticancer drugs. This was the main goal of our research.

**Figure 1:**
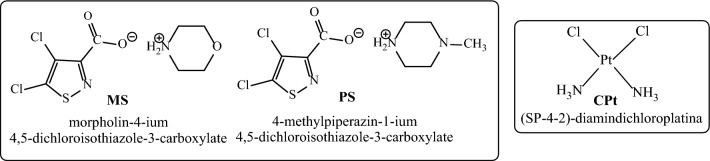
Structure of substances.

The co-authors of the article previously hypothesized that inhibitory effect on cyclin-dependent kinases (Cdk2) or VEGFR2 tyrosine kinase is one of the mechanisms for synergism of isothiazole derivatives^9^. The ability to integrate into Cdk2 sites was established by modeling with the use of the docking procedure^6^. The authors are aware that the stated explanation is only one of hypotheses that require verification and proof. In continuation of studies on the problem of synergism, this work represents a quantum-chemical analysis and modeling of “chemotherapy drug—isothiazole adjuvant” system. Cisplatin—(*SP*-4–2)-diamminedichloroplatinum (CPt), widely used in tumor therapy, was chosen as a chemotherapy drug^10^. We took available water-soluble morpholine salt of 4,5-dichloroisothiazole-3-carboxylic acid—morpholin-4-ium 4,5-dichloroisothiazole-3-carboxylate (MS) as an adjuvant, which exhibited strong synergistic effect in composition with cisplatin, as previously was established by in vitro testing on cultures of neuroepithelial tumor cells^6^. Specially synthesized analogue was taken for comparison: 4-methylpiperazin-1-ium 4,5-dichloroisothiazole-3-carboxylate (PS), which differs from MS only by the fact that morpholine fragment of the salt is replaced by an N-methylpiperazine residue (Fig. 1), which is also a pharmacophore fragment^11^.

## Experimental section

### Materials and methods

IR spectra were recorded on the Fourier spectrophotometer Thermo Nicolet Protege 460 with KBr pellets. NMR ^1^H and ^13^C spectra were registered on the Bruker Avance 500 spectrometer (500 and 125 MHz respectively) in CDCl_3_. Internal standard—residual signals of solvent CDCl_3_: 7.26 ppm for ^1^H, 77.2 ppm for ^13^C. Assignment of signals in the ^13^C NMR spectra was performed using the DEPT procedure. Elemental analysis was performed on the automatic Vario MICRO cube CHNS-analyzer. Melting points were determined on the Kofler apparatus. Monitoring of reaction progress and assessment of purity of synthesized compounds were done by TLC on Merck Millipore Silica gel 60 F254 plates, eluent Et_2_O, visualization by iodine stain.

Cisplatin was purchased from West-Ward Pharmaceuticals Corp., USA. Reagents and solvents were supplied by Aldrich Chemical Co. (China) and used without additional purification. 4,5-Dichloroisothiazole-3-carboxylic acid was prepared by the method developed by us previously^12^.

**Figure 2:**
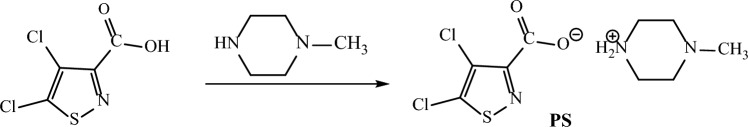
Scheme of 4-methylpiperazin-1-ium 4,5-dichloroisothiazole-3-carboxylate (PS) synthesis.

PS synthesis was carried out by 4,5-dichloroisothiazole-3-carboxylic acid reaction with N-methylpiperazine (Fig. 2), following the procedure used to prepare MS derivative^6^.

### Procedure for synthesis of PS and its spectral data

To a solution of 0.99 g (5 mmol) of 4,5-dichloroisothiazole-3-carboxylic acid in 25 ml of dry methanol, 0.50 g (5 mmol) of N-methylpiperazine was added at 20 °C, and the mixture was stirred at this temperature for 2 h. The solvent was distilled off in a vacuum to dryness, the residue was dissolved in 5 ml of dichloromethane. Then hexane was added in portions (7 × 5 ml) with stirring—after that an oil dropped out. The solvent was distilled off slowly under reduced pressure to 5 ml and the oil passed into a solid precipitate, which was filtered off, washed with hexane and dried in a vacuum. Yield 1.44 g (97%), white solid, m.p. 119–120 °C.

The structure of the obtained 4-methylpiperazine 4,5-dichloroisothiazole-3-carboxylate (PS) was confirmed by the data of IR, ^1^H and ^13^C NMR spectra and elemental analysis.

IR spectrum, ν, cm^–1^: 2991, 2974, 2948, 2851, 2803, 2770, 2718, 2632, 2468, 1635, 1574, 1454, 1425, 1394, 1342, 1312, 1283, 1192, 1145, 1098, 1082, 1048, 1003, 966, 846, 825, 799, 732, 600, 444.

NMR ^1^H spectrum (500 MHz, CDCl_3_), δ, ppm: 2.26 s (3H, CH_3_), 2.62 t (4H, 2CH_2_NCH_3_, *J* = 4.7 Hz), 3.18 t (4H, 2CH_2_NH_2_^+^, *J* = 4.9 Hz), 9.86 b.s. (2H, NH_2_^+^).

NMR ^13^C spectrum (125 MHz, CDCl_3_), δ, м.д.: 43.33 (2CH_2_NH_2_^+^), 45.90 (CH_3_), 51.71 (2CH_2_NCH_3_), 124.03, 148.48, 162.49, 165.18 (4C_quat._).

Elemental analysis. Calculated for C_9_H_13_Cl_2_N_3_O_2_S, %: C 36.25, H 4.39, N 14.09, S 10.75. Found, %: C 36.19, H 4.50, N 14.11, S 10.68.

### MTT testing

C6 rat glioma cells have been acquired from the Institute of Cytology of the Russian Academy of Sciences, Saint Petersburg (http://www.cytspb.rssi.ru, http://www.incras.ru). Determination of proliferative activity of rat C6 glioma cells after addition of cisplatin, new PS compound, and their binary mixture was carried out using MTT test by assessing optical density of medium, which is directly proportional to the number of living cells. The 3-(4,5-dimethylthiazol-2-yl)-2,5-diphenyl-2H-tetrazolium bromide (MTT) assay is widely used for determination of cell viability and proliferation. MTT, a yellow tetrazole, was reduced to purple formazan in living cells. We strictly adhered to the Merck MTT protocol (link attached) https://www.sigmaaldrich.com/RO/en/technical-documents/protocol/cell-culture-and-cell-culture-analysis/cell-counting-and-health-analysis/cell-proliferation-kit-i-mtt.

Studies were carried out in a Labconco laminar (BioHazard, USA) on C6 glioma cells in 96-well plates, the Vybrant MTT Cell Proliferation Assay Kit (Thermo Fisher Scientific, Lithuania) was used, and four series of experiments were performed. The contents absorbance of the wells was measured on an automatic biochemical immuno-fermental analyzer ChemWell^®^ 2910 (Combi) using ChemWell^®^ software version 6.3 (Revision A), USA. The initial concentration of C6 glioma cells was 5000 cells per a well of the plate. The cells were cultured in F10 nutrient medium. We added 10% bovine fetal serum to the culture medium.

Cisplatin was added at the dose of 0.3 g/m^2^, PS was added at the dose of 50 μL/ml, as well as in 10 and 100-fold dilutions (0.1 CPt, 0.1 PS and 0.01 Cpt, 0.01 PS, respectively). The effect of both individual compounds and their combinations in different ratios was determined.

### Quantum chemical methods and modeling procedures

The Gaussian 16 package software was used for the estimation of CPt-MS and CPt-PS conjugates^13^. Preliminary full geometry optimizations of initial structures were performed with the aid of molecular mechanics (MM) by using the same software. All calculations presented in this work were carried out by means of Density Functional Theory (DFT) implemented in Gaussian 16 package too^13^. Geometry optimizations of all molecules were fully optimized at the hybrid exchange–correlation functional with the Coulomb-attenuating method (CAM-B3LYP) used in our DFT calculations. CAM-B3LYP functional combines hybrid qualities of B3LYP^14,15^ and the long-range correction presented by Tawada et al.^16^. This functional works well for covalent and noncovalent, weak dispersion and hydrogen bonding was specifically developed for accurate description of long range weakly interacting systems^17,18^. Dunning's correlation consistent polarized valence double zeta basis set with adding diffuse functions aug-cc-pVDZ was used. Diffuse functions were added for describing long-range interactions such as Van der Waals forces and noncovalent interactions such as hydrogen bonding^19–21^. The LANL2DZ (Los Alamos National Laboratory 2 double zeta) basis set with effective core potential (ECP) was used for Pt^21,22^.

Polarizable continuum model (PCM, solvent is considered as continuous dielectric medium) was used for solvent phase calculations^23^. The PCM model implements self-consistent reaction field (SCRF) approach and defines solvent polarization in terms of electrostatic potential. Following discussions are based on this method if not noted otherwise. No symmetric constraints were imposed during geometrical optimizations. The energy minima were identified by subsequent frequency calculations. For visualization of quantum chemistry computations Gauss View 6.0.16 and ChemCraft software were used^13,24^.

Calculations of MEP distribution diagrams were performed using DFT / CAM-B3LYP / aug-cc-pVDZ / LANL2DZ(Pt) theory level for optimized CPt-MS and CPt-PS structures.

## Results and discussion

**Figure 3:**
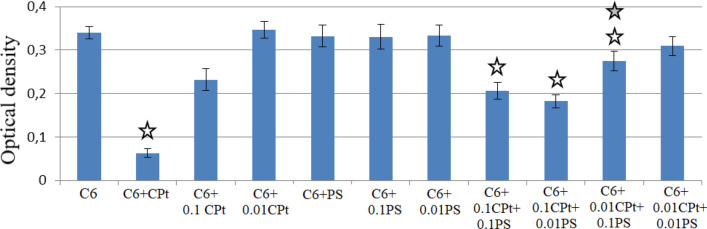
Optical density of C6 cells culture with addition of cisplatin (CPt) at different concentrations and after subtracting the background value (optical density of DMSO).

Obtained results of MTT testing of PS substance, its conjugate CPt-PS and description of the experiment are given in Table 1 in the form of numerical values and are presented in the form of diagrams (Fig. 3). As can be seen, death of tumor cells was about 83% at the dose of cisplatin 0.3 g/m^2^. tenfold reduction in dose lead to decrease up to 33%, and at 100-fold dilution cisplatin lost its antitumor effect. Individual new isothiazole derivative PS itself in all tested doses did not show any noticeable bioactivity under experimental conditions, but increased cytotoxic effect of cisplatin, although observed effect was lower than for the morpholine MS salt the test results of which were published by us earlier^6^. Thus, addition of 5 μg/ml of adjuvant (0.01 PS) to a 100-fold dilution of cisplatin (0.01 CPt), when it did not exhibit a cytotoxic effect itself, lead to noticeable antiproliferative activity and death of C6 glioma cells—up to 18%. We found that the effect of adjuvant addition to cisplatin taken in tenfold dilution (0.1 CPt) increased with decreasing dose (quite surprising!), and the cell death for combination 0.1 CPt + 0.1 PS is 38%, and for 0.1 CPt + 0.01 PS it increased up to 48%, which is almost one and a half times higher than for individual cisplatin at such dosage (32%).

**Table 1:** Optical density of C6 cell culture under the action of cisplatin (CPt), an adjuvant (PS) and their binary mixture at different concentrations and after subtracting the background value (optical density of DMSO).

	C6	C6 + CPt	C6 + 0.1 CPt	C6 + 0.01 CPt	C6 + PS	C6 + 0.1 PS	C6 + 0.01 PS	C6 + 0.1 CPt + 0.1 PS	C6 + 0.1 CPt + 0.01 PS	C6 + 0.01 CPt + 0.1 PS	C6 + 0.01 CPt + 0.01 PS
Mean	0.34	0.06	0.23	0.35	0.33	0.33	0.33	0.21	0.18	0.28	0.31
error of mean	0.01	0.01	0.02	0.02	0.03	0.03	0.02	0.02	0.01	0.02	0.02

Since the adjuvants (MS and PS) were used in doses when they themselves did not exhibit cytotoxic action, we assumed that synergistic effect appeared due to conjugation of cisplatin with isothiazole derivative and subsequent antitumor effect of molecules of resulting conjugate.

In this regard, it would be very informative to determine structural and electronic changes that occur in the system of two conjugated molecules. The information can be obtained as a result of correct quantum chemical calculations. Taking into account chemical and structural features of cisplatin and adjuvants, we assumed that association of their molecules occurred due to non-covalent interactions. To assess structure and strength of cisplatin and adjuvant complexes, as well as their physicochemical characteristics, it is correct to use methods of quantum chemistry, adapted for calculating forces of intermolecular interaction. That, DFT/CAM-B3LYP/aug-cc-pVDZ /LanL2DZ(Pt) level of theory was used in our justifications.

**Figure 4:**
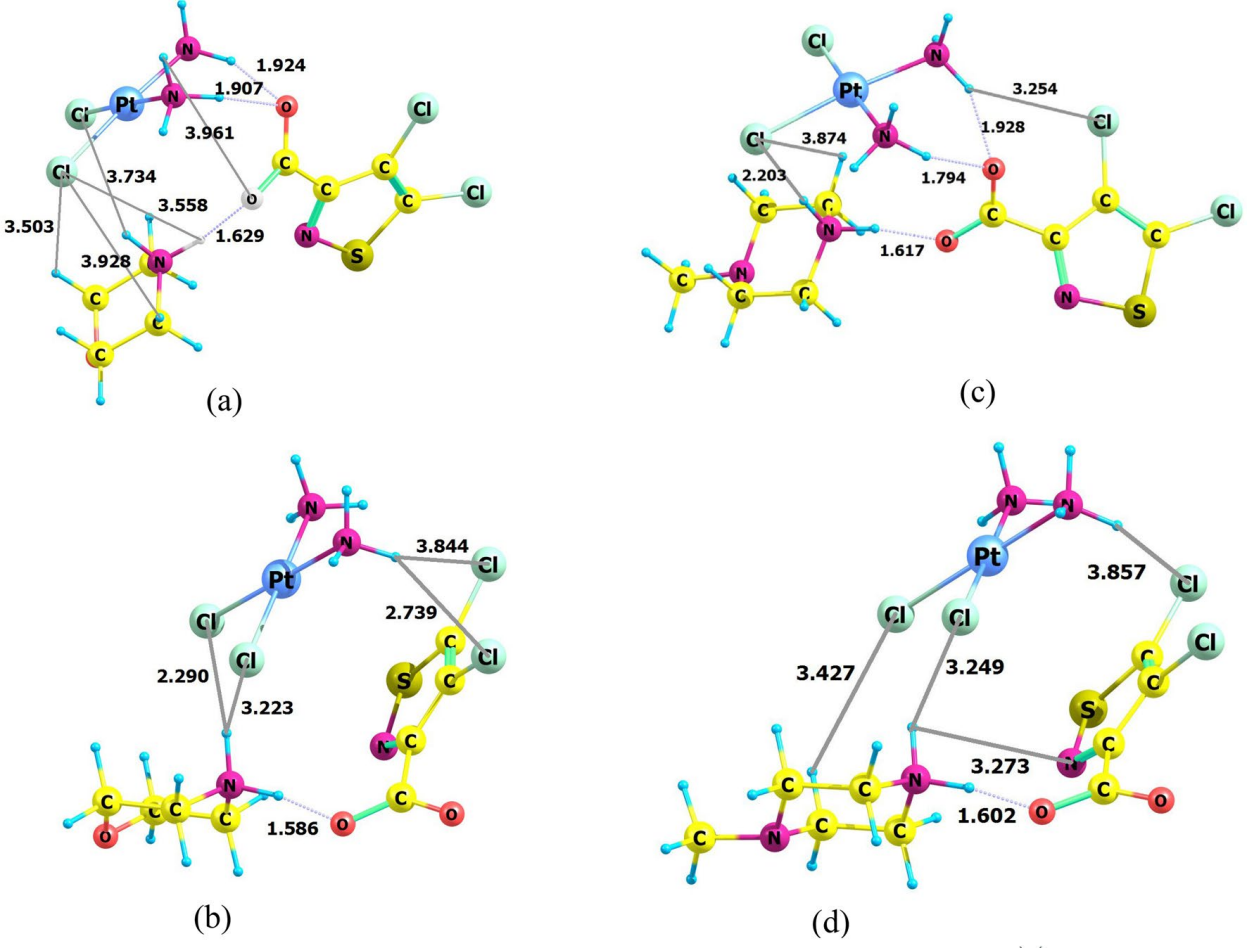
Calculated optimized structures CPt-MS (**a**,**b**) and CPt-PS (**c**,**d**) in vacuum (**a**,**c**) and with consideration to aqueous medium (**b**,**d**).

We calculated the optimal geometry of molecules, dipole moment, charge distribution, localization and energy characteristics of frontier molecular orbitals (FMO). Calculations were carried out both for individual compounds and their conjugates in two versions: isolated molecules in vacuum and with consideration to aqueous medium, which simulates situation in living cells. Molecular structures (Fig. 4) were obtained as a result of calculations with full optimization of all geometric parameters.

**Figure 5:**
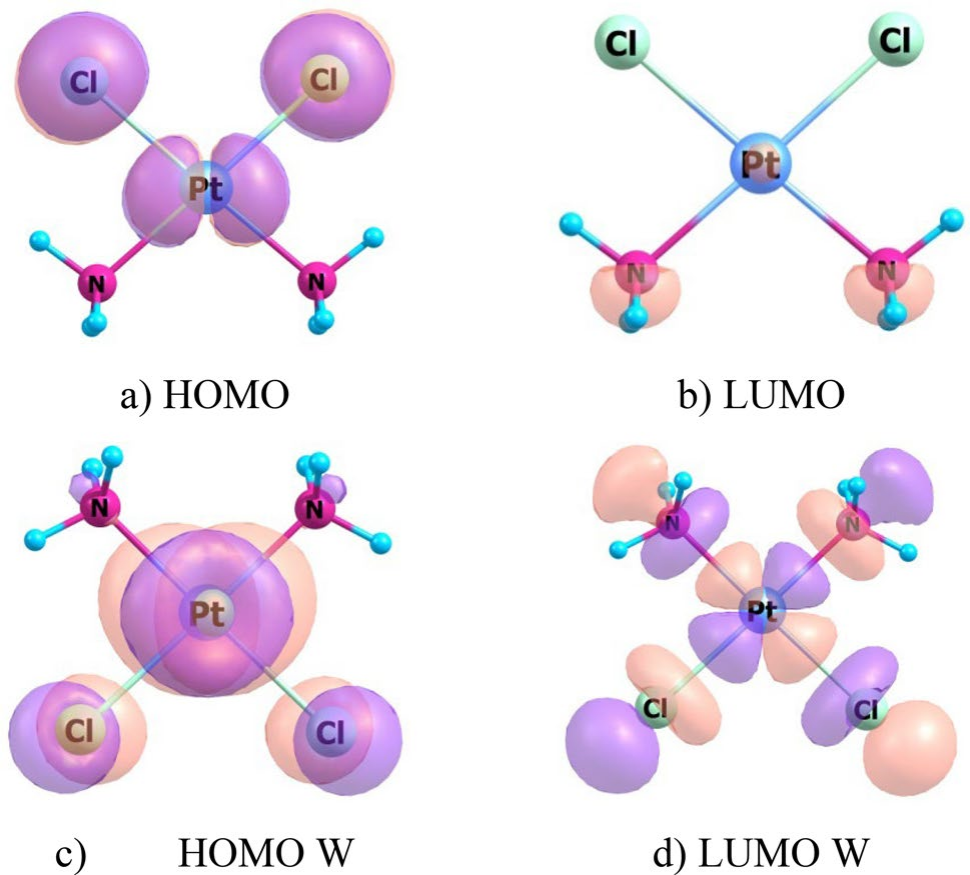
Localizations of HOMO and LUMO in cisplatin (CPt) molecule in the form of 3D isosurfaces in vacuum (**a**,**b**) and with consideration to water (**c**,**d**).

**Figure 6:**
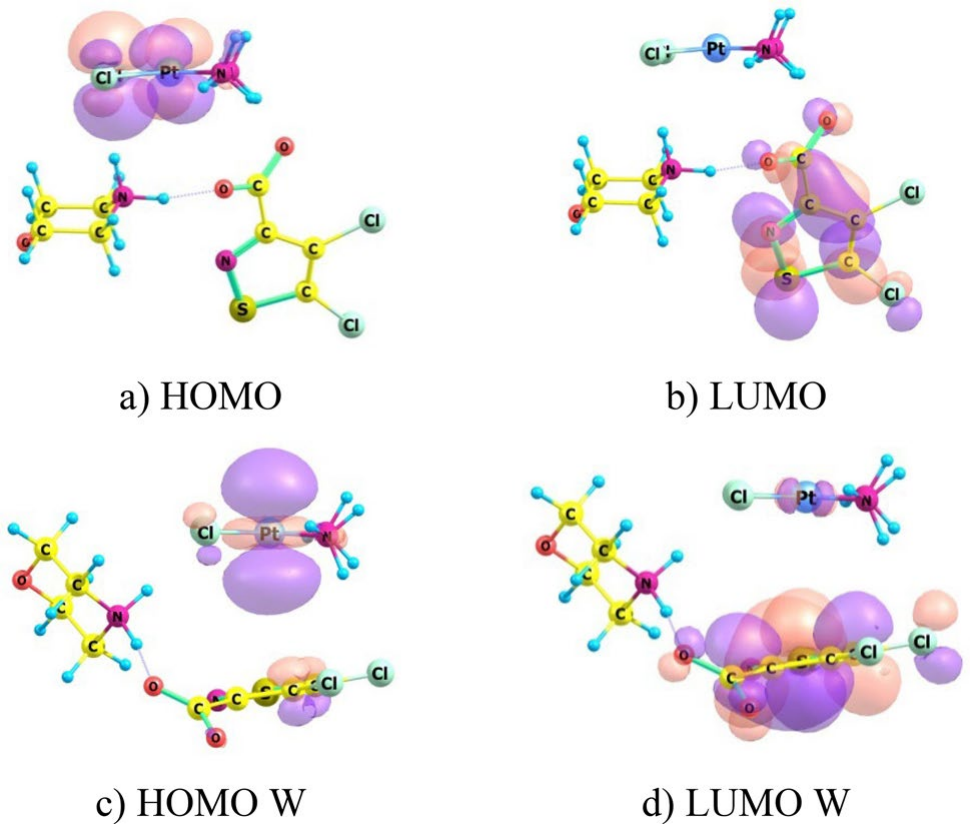
Localizations of HOMO and LUMO in molecule CPt-MS conjugate in the form of 3D isosurfaces in vacuum (**a**,**b**) and with consideration to water (**c**,**d**).

**Figure 7:**
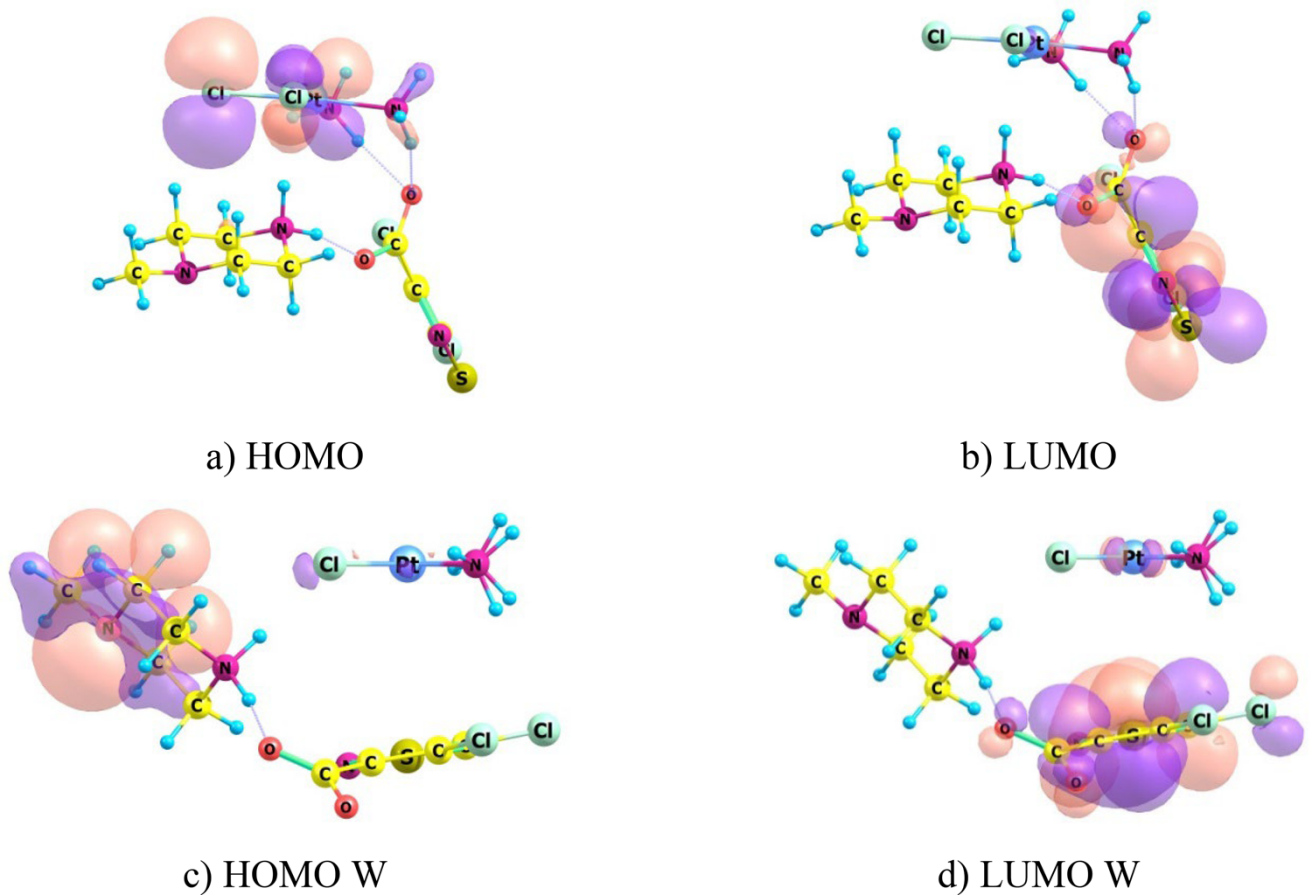
Localizations of HOMO and LUMO in molecule CPt-PS conjugate in the form of 3D isosurfaces in vacuum (**a**,**b**) and with consideration to water (**c**,**d**).

Tribak and colleagues have used data of quantum-chemical calculations of electronic structure of compounds to analyze biological activity of these substances^25,26^. Biological activity was considered by analogy with chemical activity of molecules. We also tried to apply results of calculations of optimized CPt-MS and CPt-PS structures to interpret the effect of adjuvant and elucidate possible causes of synergism in conjugate “cisplatin—isothiazole derivative”. We calculated distribution of frontier molecular orbitals (FMO): Highest Occupied Molecular Orbital (HOMO) and Lowest Unoccupied Molecular Orbital (LUMO) for CPt, CPt-MS and CPt-PS in vacuum and with consideration to aqueous medium. Results of calculating localization of HOMO and LUMO are shown in the form of 3D isosurfaces (Figs. 5, 6, 7). Determination of localization of FMO is important for establishing preferred directions and regions of the molecule for attack by nucleophiles and electrophiles. These are molecular fragments of antitumor agent that interact with target sites as applied to interpretation of biological activity.

First of all, it should be noted that the results of calculations for isolated molecules in vacuum and in an aqueous medium differ significantly. It follows from calculation data that cisplatin molecules (CPt) and isothiazole derivatives (MS and PS) form conjugates with shortened interatomic distances due to non-covalent interactions caused by hydrogen and van der Waals bonds (Fig. 4). Hydrogen bonds are clearly expressed between oxygen atom of isothiazole carboxylate residue and amine fragment of cisplatin C=O_MS/PS_···H-N_CPt_ with interatomic distances of 1.79–1.93 Å in vacuum in both conjugates. These connections are absent in aquatic environment. There are some other interplays in structures of conjugates that differ in aqueous medium and vacuum. For example, for both complexes, shortened contacts are observed between chlorine atoms of cisplatin and CH fragments of morpholine and piperazine (Cl_CPt_···H-C_morph_, Cl_CPt_···H-C_pip_), which values in aqueous medium (2.29, 3.22, 3.43 Å) are less than in vacuum (3.50, 3.87 Å), and corresponding values for CPt-PS conjugate are higher than for CPt-MS conjugate. However, there is no common single trend in the change in interatomic distances between the corresponding molecular fragments during the transition from vacuum to an aqueous medium. In particular, for CPt-PS conjugate, the Cl_isoth_···H-N_CPt_ and Cl_CPt_···H-N_pip_ values in vacuum are lower than in aqueous medium, in contrast to Cl_CPt_···H-C_pip_ values. It can only be stated with confidence that mutual arrangement of molecules of heterocycles (MS and PS) and cisplatin (CPt) and their relative orientation in vacuum and in aqueous medium differ significantly. The differences found are of great importance for interpretation and modeling of antitumor effect of substances, since the agent-target interaction in reality takes place in aquatic environment.

Both in vacuum and aqueous medium HOMO is localized on Pt and Cl atoms in individual cisplatin molecule CPt (Fig. 5a,c). LUMO is localized on NH_3_ groups in vacuum (Fig. 5b) and delocalized throughout the molecule in aqueous medium (Fig. 5d). It is important to know what happens to its molecule when conjugated with adjuvant. It turns out that LUMO is localized on isothiazole heterocycle in both CPt-MS and CPt-PS conjugates in vacuum and aqueous medium. HOMO is localized in CPt-MS conjugate, both in vacuum and aqueous medium, on cisplatin molecule with maximum density on Cl–Pt–Cl fragment, same as in individual cisplatin molecule, but the contribution of Pt atom in aqueous medium in CPt-MS is greater than in individual cisplatin. HOMO is also located on Cl–Pt–Cl fragment in CPt-PS conjugate in vacuum. The picture fundamentally changes in aquatic medium—HOMO is localized on piperazine fragment.

For explanation of results obtained the mechanism of cytotoxic action of cisplatin was taken into account. Despite the differences in interpretation of some precise details, it is believed that cytotoxic effect of cisplatin is due to disruption of DNA functions via binding to purine bases in its molecule. This leads to intra- and inter-stranded DNA cross-linking, which causes DNA damage, impaired replication and transcription, and subsequently apoptosis of cancer cells^27–29^.

Localization of HOMO and LUMO determines which fragments of the molecule are preferred for attack by electrophiles and nucleophiles, respectively. The following conclusions can be drawn from the analysis of FMO distribution. Formation of CPt-MS and CPt-PS conjugates leads to displacement of LUMO from cisplatin molecule and its localization on isothiazole heterocycles both in vacuum and aqueous medium. Since LUMO determines interaction with target nucleophilic sites, this type of binding will be realized through isothiazole heterocycle and not through cisplatin molecule. With regard to generally accepted mechanism, we can say that binding to purine bases in CPt-MS and CPt-PS conjugates will proceed with direct participation of adjuvant, which explains its role in manifestation of synergistic effect.

Cross-linking of DNA strands can cause local denaturation and damage to DNA and appearance of new sites, including electrophilic ones. Binding to electrophilic DNA sites determines localization of HOMO in conjugate. Calculations for conjugate molecules in vacuum showed that HOMO in both conjugates is completely localized on cisplatin molecule. Calculations with consideration to aquatic medium revealed differences in localization of HOMO in conjugates. It still remains on cisplatin molecule in CPt-MS, while in CPt-PS it is predominantly localized on PS piperazine residue. This may be one of the reasons for lower activity of CPt-PS compared to CPt-MS.

The energies of frontier molecular orbitals (FMO) are also used as one of characteristics for biological activity of a molecule. The key characteristics of a molecule in FMO theory are the difference between the energies of HOMO and LUMO (ΔE), global hardness and softness of the system (η and S) which are treated as descriptors. We performed calculation of descriptors as well as dipole moments for optimized structures CPt, CPt-MS and CPt-PS in vacuum and with consideration to aqueous medium. The obtained values are shown in Table 2.

**Table 2:** Calculated DFT method values of descriptors ΔE, η and S and dipole moments in vacuum and in aqueous medium for CPt, CPt-MS and CPT-PS.

Descriptors	In vacuum			In aqueous medium		
	CPt	CPt-MS	CPt-PS	CPt	CPt-MS	CPt-PS
LUMO (eV)	−0.7946	−0.7215	−0.4042	−0.3053	−0.5667	−0.5550
HOMO (eV)	−7.8982	−8.0759	−8.0815	−8.2420	−8.2638	−8.0346
ΔE (eV)	7.1035	7.3544	7.6773	7.9367	7.6971	7.4796
η	3.5518	3.6772	3.8386	3.9683	3.8486	3.7398
S	0.1408	0.1360	0.1303	0.1260	0.1299	0.1337
Dipole moment (Debye)	10.954	3.271	2.502	16.438	19.032	18.990

The differences in calculated descriptors’ values for CPt, CPt-MS and CPt-PS are small. However, there is a tendency to decrease in ΔE when going from CPt to conjugates in calculations with consideration to aqueous medium. This indicates an increase in reactivity of the system, which in our case means more active binding with DNA. There is a slight decrease in global hardness and a small increase in global softness in CPt—CPt-MS—CPt-PS series. It should be noted that the opposite situation is observed for calculations in vacuum.

The difference in values of dipole moments is very significant, and in vacuum the dipole moment of conjugates is greatly reduced in comparison with cisplatin (3.27, 2.50 and 10.95 D respectively), but in aqueous medium, on the contrary, the dipole moment of conjugates is higher than that of cisplatin (19.03, 18.99 and 16.44 respectively), which indicates a greater polarity of conjugate molecules. We have noted such differences in the data for isolated molecules in vacuum and aqueous medium during FMO analysis. This represents great influence of environment (water) on structure and properties of the objects under consideration.

**Figure 8:**
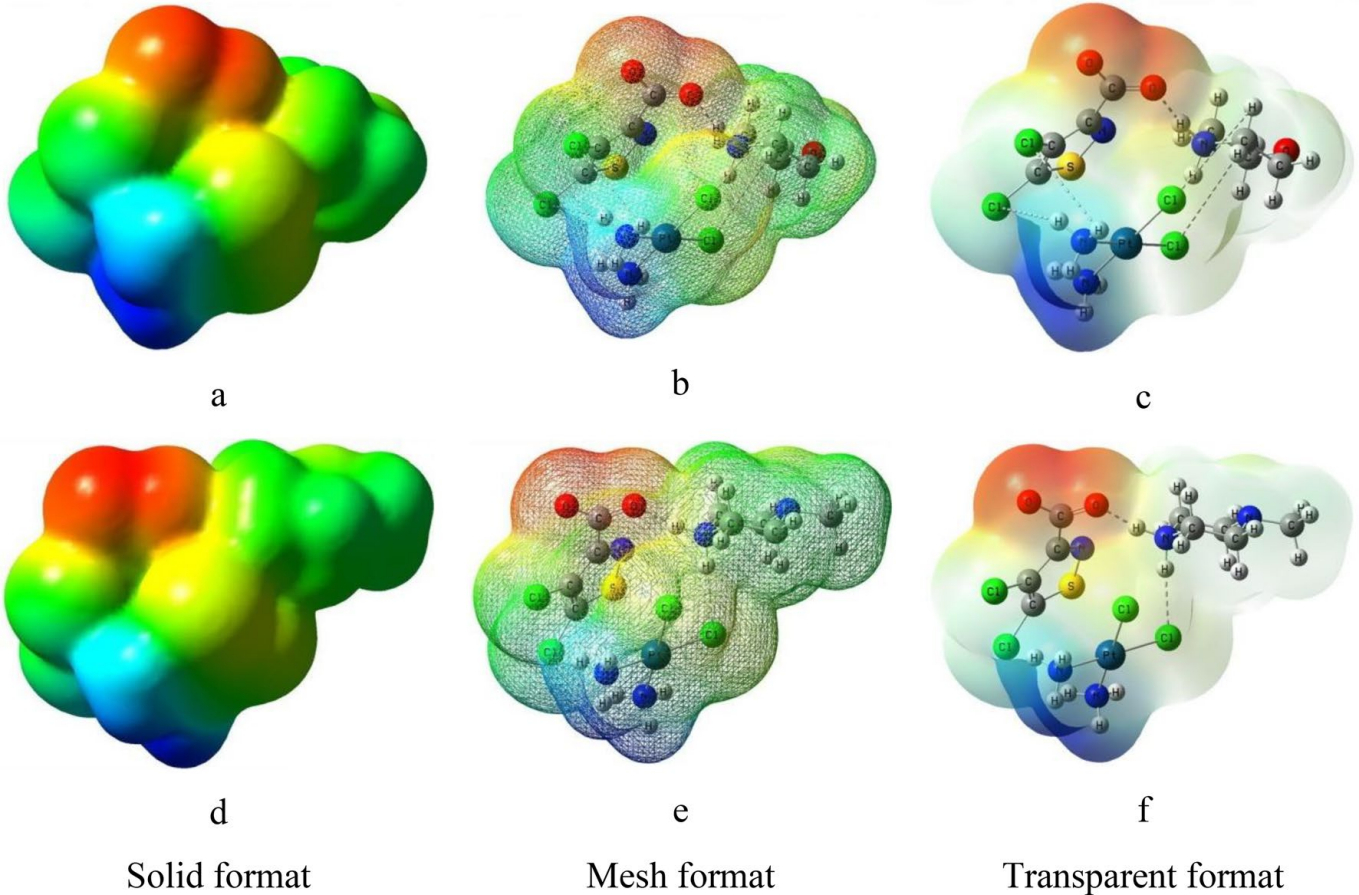
Molecular electrostatic potential diagram of CPt-MS (**a**–**c**) CPt-PS (**d**–**f**) in different formats.

We used one more descriptor for the analysis of “cisplatin – isothiazole derivative” system – molecular electrostatic potential (MEP)^30^. It allows evaluating electrostatic component of the energy of intermolecular interactions and is clearly presented in the form of color diagrams. The MEP chart can be used to predict reactivity and active sites for interactions. Negative electrostatic potential corresponds to proton attraction by total electron density in a molecule, that is, protonation of a molecule (shades of red), and positive electrostatic potential corresponds to proton repulsion by atoms (shades of blue). The potential increases in the following order: red < orange < yellow < green < blue. The MEP distribution diagrams in different formats calculated with consideration of aquatic environment are shown in Fig. 8. The calculation results show that regions with negative potential are located on oxygen atoms of carboxyl group (–O–C=O) of isothiazole derivative. Areas with positive potential are localized around amino groups of cisplatin. Thus, conjugation of adjuvant with cisplatin results in formation of molecular substrate in which types of electrostatic interactions are shared between cisplatin and isothiazole ligand.

## Conclusions

In general, the following conclusions can be drawn from the obtained results.

The results of quantum-chemical calculations of the optimal geometry and electronic structure of molecules differ for isolated molecules in vacuum and in an aqueous medium and allow revealing one of possible reasons for the effect of adjuvant in combined chemotherapy of tumors, namely:

1. Adjuvants of isothiazole series shape conjugates with cisplatin with non-covalent interactions between molecules due to hydrogen, van der Waals and electrostatic bonds. The resulting conjugate of two molecules acts as a single unit.
2. Formation of conjugates leads to relocation of frontier molecular orbitals between cisplatin molecules and adjuvant. LUMO localizes on isothiazole heterocycle, which appears to be preferred for binding with DNA molecules. This fact allows explaining the effect of adjuvant in used composition.
3. Unlike LUMO, HOMO is localized on cisplatin molecule in CPt-MS conjugate, while in CPt-PS conjugate it is located on cisplatin molecule in vacuum and mainly on piperazine PS fragment in aqueous medium. These features are one of the reasons for lower activity of CPt-PS compared to CPt-MS.
4. Convergence of energies of frontier molecular orbitals, small changes in global hardness indicate increase in reactivity of conjugates due to association of cisplatin with adjuvant, which is accompanied by increase in cytotoxic effect.
5. Increase in the dipole moment of conjugates in aqueous medium, i.e., polarity of their molecules, in comparison with individual cisplatin, also promotes activation of cytotoxic agent binding to the target, which enhances manifestation of adjuvant effect.
6. Diagrams of MEP distribution in conjugates demonstrate significant role of isothiazole ligand in distribution of electrostatic potential in the system.

Thus, the results of quantum-chemical calculations revealed various aspects of isothiazole adjuvant effect in conjugated state with cisplatin on manifestation of cytotoxic activity against tumor cells. We are guided by the concept of using different adequate approaches and synergistic effects to achieve the positive results in treatment of malignant neoplasms^31,32^. Implementation of proposed methodology in the process of experimental research in clinical practice is able to turn hypothesis into reality.

The data obtained in experimental studies are the basis for expanding the scope of such studies to include other chemotherapy drugs that are used in modern oncology, as well as developments with the study of other adjuvants’ effects. Such tactics will result in expansion of indications for the use of chemotherapy drugs recommended by international protocols in reduced dosages. Oncologists will have opportunity to use “classical” chemotherapy drugs in combination with synergists for those patients who have not previously been recommended to such prescriptions because of pronounced toxic side effects. Moreover, there is an obvious economic effect of using “classical” chemotherapy drugs in comparison with expensive procedure for development, testing and introduction of new chemotherapeutics.

## Data Availability

Biotesting data are in body text. An array of data obtained as a result of quantum chemical calculations and modeling is available at the link https://www.bsuir.by/m/12_100229_1_160639.zip.
